# Prebiotic Potential of Culinary Spices Used to Support Digestion and Bioabsorption

**DOI:** 10.1155/2019/8973704

**Published:** 2019-06-02

**Authors:** Christine T. Peterson, Dmitry A. Rodionov, Stanislav N. Iablokov, Meredith A. Pung, Deepak Chopra, Paul J. Mills, Scott N. Peterson

**Affiliations:** ^1^UC San Diego, School of Medicine, Center of Excellence for Research and Training in Integrative Health, Department of Family Medicine and Public Health, La Jolla, CA, USA; ^2^Sanford Burnham Prebys Medical Discovery Institute, Bioinformatics and Structural Biology Program, La Jolla, CA, USA; ^3^Institute for Information Transmission Problems, Russian Academy of Sciences, Moscow, Russia; ^4^P.G. Demidov Yaroslavl State University, Yaroslavl, Russia; ^5^Chopra Foundation, Department of Ayurveda and Yoga Research, Carlsbad, CA, USA; ^6^Sanford Burnham Prebys Medical Discovery Institute, Tumor Microenvironment and Cancer Immunology Program, La Jolla, CA, USA

## Abstract

Although the impact of medicinal and culinary herbs on health and disease has been studied to varying extents, scarcely little is known about the impact of these herbs on gut microbiota and how such effects might contribute to their health benefits. We applied* in vitro *anaerobic cultivation of human fecal microbiota followed by 16S rRNA sequencing to study the modulatory effects of 4 culinary spices:* Curcuma longa* (turmeric),* Zingiber officinale* (ginger),* Piper longum* (pipli or long pepper), and* Piper nigrum* (black pepper). All herbs analyzed possessed substantial power to modulate fecal bacterial communities to include potential prebiotic and beneficial repressive effects. We additionally analyzed the sugar composition of each herb by mass spectrometry and conducted genome reconstruction of 11 relevant sugar utilization pathways, glycosyl hydrolase gene representation, and both butyrate and propionate biosynthesis potential to facilitate our ability to functionally interpret microbiota profiles. Results indicated that sugar composition is not predictive of the taxa responding to each herb; however, glycosyl hydrolase gene representation is strongly modulated by each herb, suggesting that polysaccharide substrates present in herbs provide selective potential on gut communities. Additionally, we conclude that catabolism of herbs by gut communities primarily involves sugar fermentation at the expense of amino acid metabolism. Among the herbs analyzed, only turmeric induced changes in community composition that are predicted to increase butyrate-producing taxa. Our data suggests that substrates present in culinary spices may drive beneficial alterations in gut communities thereby altering their collective metabolism to contribute to the salubrious effects on digestive efficiency and health. These results support the potential value of further investigations in human subjects to delineate whether the metabolism of these herbs contributes to documented and yet to be discovered health benefits.

## 1. Introduction

Digestive disorders are increasingly prevalent in Western populations with over 60 million people affected in the United States alone [1]. Integrative and traditional medicine practices focus on proper digestion and absorption of food through the administration of medicinal herbs that are taken with food and used as culinary spices in food preparation to stimulate digestive processes. Proposed mechanisms of action for digestive stimulation by spices include increased bile production and enhanced digestive enzyme production to include salivary, gastric, biliary, pancreatic, and terminal small intestine mucosal secretions [2]. The modulation of the structure and function of gut microbiota is likely another mechanism through which culinary spices stimulate digestive functions and was thus the focus of the current investigation.

Culinary spices are derived from various plant parts, such as roots, leaves, fruits, stems, rhizomes, seeds, and bark, and contain phytochemical constituents that are of interest for their medicinal value. Indeed, investigations of culinary spices have revealed potent anti-inflammatory, antimicrobial, antidiabetic, and antimutagenic activities [3–7]. While indirect prebiotic effects from spice antioxidant constituents may contribute to the effect, direct prebiotic effects of spice carbohydrate and amino acids on gut microbiota communities have been scarcely been studied [4, 8, 9]. Human microbiota investigations by our group have revealed the significant prebiotic potential of medicinal herbs used in digestive health and disease via the microbial metabolism of herb-provided substrates, e.g., sugars, glycans and amino acids [10]. Here we apply these approaches to a set of culinary herbs to expand our understanding of potential prebiotic effects of these herbs compared to those previously studied by our group and others [11]. The gut microbiota encodes untold biotransformation potential of phytochemicals, exemplified by microbiota-dependent bioconversion of polyphenolic compounds that serve to increase their absorption and bioactivity, including anti-inflammatory compounds [12–14]. Both fibers and phytochemicals in medicinal herbs used as spices appear to promote the growth of beneficial microbes and inhibit the growth of potentially inflammatory species [15].

The 4 medicinal herbs selected for investigation in the current study are widely used therapeutically in traditional and integrative medicine for both digestive disorders and other diseases as well as for the promotion of proper digestion and absorption of food. These herbal medicines are also culinary spices used to prepare foods and included* Curcuma longa* (common name: turmeric),* Zingiber officinale* (common name: ginger),* Piper longum* (common name: pipli or long pepper), and* Piper nigrum* (common name: black pepper) (Table 1). For example, Ayurveda, the traditional system of medicine in India, often includes these herbal medicines in spice formulations that are taken with food or cooked into food to enhance digestive capacity and absorption of nutrients. Ginger, black pepper, and pipli are often combined in equal quantities in the common Ayurvedic Medicine formulation called* Trikatu*, meaning three acrids, which is used as a bioavailability enhancer with its main action deriving from the piperine alkaloid [16]. These culinary spices likely interact with gut microbiota to induce gastrointestinal and systemic effects.

The 4 culinary spices we investigated contain constituents that have medicinal value such as polyphenols, which are known to be metabolized by gut microbiota [12]. Ginger, commonly used for its antimicrobial, bioabsorption, and anti-inflammatory effects, contains several identified bioactive constituents including gingerol and gingerol-like compounds and antioxidants such as beta-carotene, ascorbic acid, polyphenols, terpenoids, and alkaloids [17–19]. Both examined members of the* Piper* genus contain terpenes, antioxidants, B vitamins, and alkaloids such as piperine and piperine-like compounds and have been used medicinally for their anti-inflammatory, antiasthmatic, analgesic, antiepileptic, chemopreventative, and antihelminthic effects [20, 21]. Both black pepper and pipli biotransform increase the bioavailability of many food, drug, and phytochemical substrates [22].

Turmeric is well-studied and commonly used for its antimicrobial, antioxidant, and anti-inflammatory action in a variety of applications including gastrointestinal, dermatological, and neurological disorders [23]. The main bioactive compounds in turmeric are curcuminoids, which contain curcumin, demethoxycurcumin, and bisdemethoxycurcumin, with limited efficacy due to low bioavailability [24]. Other turmeric constituents include diaryl heptanoids, turmerones, diarylpentanoids, phenylpropenes, monoterpenes, sesquiterpenes, diterpenes, triterpenoids, sterols, and various alkaloids [25]. Curcumin is of high interest due to its anti-carcinogenic effects potentially mediated by inducing apoptosis, inhibiting cell cycle, and positive modulatory effects on the microbiome [26]. However, the impact of these herb constituents on gut microbiota and the microbial metabolism of these spices and their constituents remain incompletely understood.

The culinary spices of interest display both increases and specific inhibitory effects. Ginger exhibits* in vitro* antibacterial activity towards inflammatory gut species such as* Escherichia coli* and* Klebsiella pneumonia* [27]. Both ginger and turmeric enhance the growth of beneficial* Bifidobacterium* spp. and* Lactobacillus* spp. while repressing the growth of several* Ruminococcus* spp. derived from clinical samples [4]. In a porcine intervention, beneficial* Bacteroides intestinalis*,* Eubacterium oxidoreducens*,* Selenomonas* spp., and* Phascolarctobacterium faecium* were increased in abundance in animals supplemented with ginger while abundance of pathogenic* Atopostipes suicloacalis* and* Bartonella quintana* were decreased compared to controls [15].* Salmonella enterica* growth was inhibited by black pepper* in vitro*; however, little is known about the effects of pipli on gut microbiota [28].* In vitro*, turmeric increased* Ruminococcus* spp. and a few* Clostridium* isolates but did not affect beneficial* Lactobacillus* spp. or* Bifidobacterium* spp. [4]. In a pilot human clinical trial, turmeric treatment increased the relative abundance of most* Clostridium* spp.,* Bacteroides* spp.,* Citrobacter* spp.,* Cronobacter* spp.,* Enterobacter* spp.,* Enterococcus* spp.,* Klebsiella* spp.,* Parabacteroides* spp., and* Pseudomonas* spp. and reduced several* Blautia* spp. and most* Ruminococcus* spp. [8]. Thus, culinary spices may exert both prebiotic effects through positive selection and antimicrobial action. However, additional human studies are needed to support these initial findings and further understand the impact of these culinary spices and constituents on microbiota metabolic activities.

In the current investigation, anaerobic human fecal cultivation was used to investigate the extent to which 4 herbal medicines commonly used as culinary spices and for digestive health alter the growth and abundance of gut bacterial species. Human gut microbiota or their metabolites may mediate aspects of the beneficial effects of these medicinal herbs; however, scarcely little is known about the gut microbiota in the context of each culinary spice. We applied 16S rRNA sequencing of human fecal microbiota to evaluate the prebiotic potential of these medicinal herbs. To facilitate deeper interpretation of community profiles, we performed genome reconstruction of sugar utilization and short chain fatty acid (SCFA) pathways and glycosyl hydrolase (GH) families. We analyzed the sugar composition of each herb by quantitative mass spectrometry (MS) to focus these analyses on potential drivers, and their relevance. Thus, it was hypothesized that substrates present in culinary spices drive alterations in gut communities thereby altering their collective metabolism to contribute to the beneficial effects on digestive efficiency and health.

## 2. Methods

*Study participants and sample collection. * Twelve healthy, English-speaking women and men aged 30–60 years that had adhered to a vegetarian or vegan diet for >1 year were recruited to donate a single stool sample. This study was approved by and carried out in accordance with the recommendations of Sanford Burnham Prebys Medical Discovery Institute Institutional Review Board (IRB-2014-020) and guidelines with written informed consent from all subjects. All subjects gave written informed consent in accordance with the Declaration of Helsinki. Participants ate their normal diets and donated a morning fecal sample in stool hats (Fisher Scientific). The fecal samples were transferred to conical tubes and stored at -80°C until further processing.

*Digestive herbal medicines examined*. We examined 4 medicinal herbs in this study (Table 1). The turmeric, ginger, and pipli organic spice powders were sourced from Banyan Botanicals (Albuquerque, NM). The organic Tellicherry whole black peppercorns were sourced from Frontiers Co-op (Norway, IA) and ground to a fine powder in a spice grinder (Krups).

*Anaerobic fecal cultures. *Equal volumes of stool collected from 12 healthy vegetarian participants were pooled and used to inoculate (approximately 10^6^cells) a chemically defined medium (CDM) or CDM supplemented with 1% herb in Hungate tubes. These procedures have been described elsewhere [11].

*Microbial DNA Isolation. *Genomic DNA was isolated from cultures using the procedures of the QiaAmp DNA stool kit (Qiagen) with a modification that included an additional step of bead beating using the Thermo FastPrep instrument (MP Bio) to ensure uniform lysis of bacterial cells.

*16S rRNA sequence analysis*. Multiplexed 16S rRNA libraries were prepared using standard 16S metagenomic sequencing library protocols from Illumina, which uses oligonucleotides targeting the V3-V4 region of 16S rDNA for PCR amplification. Details of data analysis have been described elsewhere [11].

*Genome reconstruction of sugar metabolism and SCFA pathways.* To predict metabolic capabilities of microbial taxa identified by 16S analysis, we performed genomics-based reconstruction of metabolic subsystems including 11 subsystems involved in sugar uptake and utilization and two subsystems for SCFA synthesis implemented in the SEED genomic platform [29] to capture, analyze, and extend pathways, enzymes, and transporters involved in sugar and SCFA metabolism in >2,200 microbial genomes. Details of data analysis have been described elsewhere [11]. The obtained binary phenotype matrix (BPM) for reference genomes was used to calculate a community phenotype matrix (CPM) for all mapped taxa obtained from 16S analysis by averaging the respective CPM values (Table S3). Community phenotype index (CPI) for each 16S sample was calculated as the sum of respective CPM values of each taxa multiplied by their relative abundances. CPI gives a probabilistic estimate of a fraction of cells in the community possessing a specific metabolic pathway (on a scale 0 -100%).

*Mass spectrometry analysis of nervine herbal medicines.* All samples (1% w/v) were hydrolyzed with 2M TFA at 100°C for 4 hrs for analysis of monosaccharide analysis. Details of data analysis have been described elsewhere [11].

*Statistical analyses. *Descriptive statistics were computed in initial analyses for all variables of interest. In this pilot study, statistical assumptions of normality and variance homogeneity of variance were not met; thus the nonparametric Kruskal-Wallis test was first used to examine whether there were differences in the mean ranks of the relative abundance of each species in response to different spices. These Kruskal-Wallis tests were two-tailed, and alpha level was set at 0.05. Next, in the case of a significant omnibus test for a given species, Dunn's pairwise tests were conducted to examine the* a priori* hypotheses that the relative abundance of each species for each spice was different than control. To correct for familywise error in these planned comparisons (each of the 4 spices versus control), alpha was set at .0125 using the Bonferroni correction. All analyses were conducted using IBM SPSS Statistics for Macintosh Version 25.0 (Armonk, NY). The significance of differences in families and GH gene representation was determined using one-way ANOVA with Dunnett's correction for multiple comparisons.

## 3. Results

To examine the direct effects of herbal medicines used as culinary spices and as digestive aides in the absence of dietary and host-driven processes, we used* in vitro* anaerobic fecal cultivation of human fecal inoculums representing a pool of 12 healthy vegetarian donors. The results are presented with the understanding that gut microbes capable of catabolizing medicinal herbs will exhibit a growth advantage revealed by increased representation in the microbial community. These respective changes will be balanced by reduced relative abundance of taxa displaying reduced fitness in herb-supplemented cultures and those directly inhibited by herb constituents with antimicrobial activities. The relative abundance of gut species cultured in a chemically- defined medium (CDM) lacking carbohydrate energy sources (control, n=6) was compared to cultures grown in CDM supplemented with turmeric (n=4), ginger (n=4), black pepper (n=4), or long pepper (n=5).

**Figure 1:**
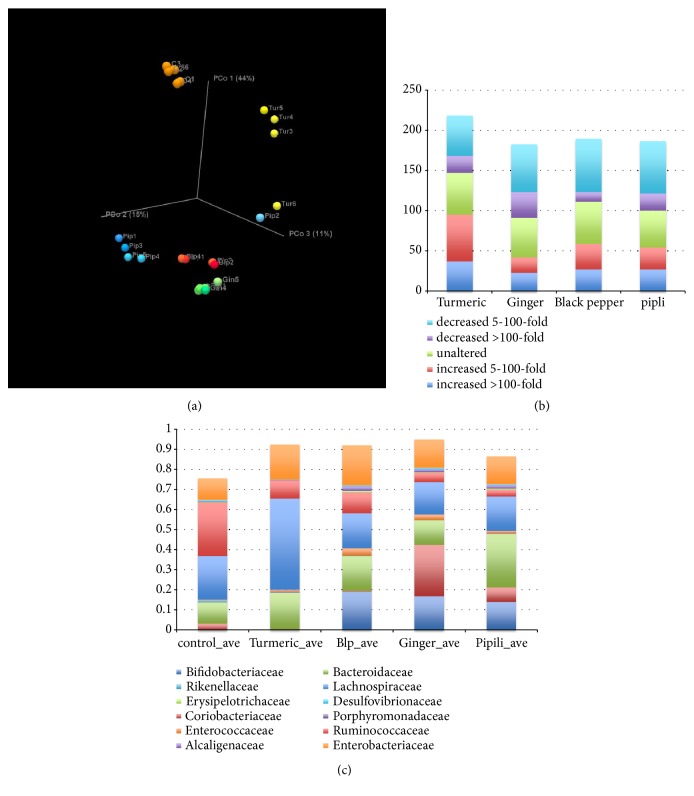
*Herb induced modulatory effects.* (a)* PCoA of nervine herbs*. Bray-Curtis PCoA beta-diversity plots of communities observed in C=control, Tur=Turmeric, Blp=Black pepper, Gin=Ginger, Pip=Pipli. (b)* Modulatory capacity of nervine medicinal herbs*. Average fold change of taxa comparing herb-supplemented to control cultures. Zeros were replaced with e^−6^ to permit minimum fold-change values to be calculated. (c)* Family representation*. Relative abundance of bacterial families in herb-supplemented cultures.

*Herb-induced alteration in fecal communities.* Collectively, 225 unique bacterial taxa (species approximation), belonging to a large number (105) of distinct genera, were observed (Table S1). Analyses were conducted to identify taxa that were statistically different (p<0.05) than control cultures (Table S1). Representation of the *β*-diversity of communities present in control and herb-supplemented cultures by PCoA suggests that each herb drives unique alterations in community composition (Figure 1(a)).

Interestingly, black pepper, pipli, and ginger supplemented cultures generated more highly related communities, whereas turmeric selected for more distinct bacterial communities. Outlier communities were observed in one case for both pipli and turmeric-supplemented cultures, a phenomenon we previously observed (PMID: 30889210), suggesting that they reflect alternative communities with potentially distinct or overlapping metabolic preferences. These alternative community configurations are predicted to possess similar community fitness to exploit herb substrates.

Comparison of the average relative abundance of species present in control cultures to those present in one or more herb-supplemented cultures revealed that all herb-supplemented cultures displayed substantial and similar modulatory power involving 72-76% of the observed taxa (Figure 1(b)). Turmeric induced the largest number of taxa increased in relative abundance, whereas ginger, turmeric, and black pepper-supplementation drove the largest number of taxa displaying decreased relative abundance.

Compared to control cultures, each herb selected unique proportions of bacterial families (Figure 1(c)). Compared to control cultures, black pepper (p<0.0001), ginger (p=0.0004), and pipli (p=0.001), but not turmeric, were Bifidogenic. Turmeric drove expansions of Bacteroidaceae (p=0.03), Desulfovibrionaceae (p=0.01), and particularly Rikenellaceae (p<0.0001) and Lachnospiraceae (p=0.0006). Black pepper supplemented cultures were enriched for Rikenellaceae (p=0.0003), Enterococcaceae (p=0.0013), Erysipelotrichaceae, Alcaligenaceae (p<0.0001), and Enterobacteriaceae (p=0.05) at the expense of Ruminococcaceae (p=0.0008). Ginger strongly induced the increased relative abundance of Coriobacteriaceae (p<0.0001), Rikenellaceae (p=0.0001), Enterococcaceae (p=0.01), Erysipelotrichaceae (p=0.005), and Alcaligenaceae (p=0.03) at the expense of Ruminococcaceae (p=0.0002). Finally, pipli enriched Porphyromonadaceae (p=0.006), Erysipelotrichaceae (p=0.0004), and Alcaligenaceae (p=0.0007) at the expense of Ruminococcaceae (p<0.0001) and Rikenellaceae (p=0.0002).

*Genus level impact of herbal medicines on gut microbiota*. Compared to the other medicinal herbs tested, turmeric induced the greatest alterations in community composition, preferentially increasing the relative abundance of* Clostridium *spp. (11 taxa), 4 of which were statistically significant (p=0.04-0.0001);* Bacteroides *spp. (9 taxa), 6 of which were statistically significant (p=0.04-0.0001);* Blautia *(3 taxa), all of which were statistically significant (p=0.005-0.001); and* Enterobacter *spp. (4 taxa), all of which were statistically significant (p=0.002-0.0001). Notably, we observed that opportunistic pathogens such as* Citrobacter freundii and Enterococcus faecalis *displayed reduced relative abundance in turmeric-supplemented cultures, whereas* Shigella dysenteriae *and* Escherichia coli *displayed reduced relative abundance in all herb cultures (Table S1).

We compared the dominant taxa (>1%, collectively 83-86% of the total community) generated by each herb (Table S2A). Among dominant taxa, turmeric induced an increase in the greatest number of unique genera compared with control cultures including representatives:* Eubacterium rectale *(p=0.001)*, Ruminococcus bromii *(p=0.002)*, Roseburia hominis *(p=0.0001)*, Bacteroides vulgatus *(p=0.003)*, Flavonifractor plautii, Gemmiger formicilis, *(p=0.03)*, Klebsiella variicola *(p=0.003), and* Blautia hydrogenotrophica *(p=0.003) and* Acidaminococcus intestine *(p=0.002). Ginger strongly selected for* Collinsella aerofaciens *(p=0.0001)*, Bifidobacterium faecale *(p=0.002),* Bacteroides xylanolyticus *(p=0.0001), and* Eubacterium eligens *(p=0.001). Common to many of these taxa is the capacity to produce short chain fatty acids (SCFAs), particularly butyrate and propionate (see below). Other taxa were dominant in all herb-supplemented cultures including* Bacteroides *spp.,* B. vulgatus, B. uniformis, B. cellulosilyticus, Phascolarctobacterium faecium, Klebsiella *spp.,* K. variicola *and* K. pneumoniae*, and* Oscillibacter ruminantium; *however statistically significant differences were not always observed due to high relative abundance in control cultures also. 

A total of 30 taxa were increased in relative abundance relative to control cultures for all medicinal herbs tested (Table S2B). It should be noted that the majority of these taxa were present in very low abundance in control cultures. These taxa are phylogenetically diverse, featuring enrichment of three* Bacteroides *spp.,* B. dorei, B. vulgatus, *and* B. xylanolyticus *and three* Klebsiella *spp. Each herb altered a large number of genera with limited species representation. This signature is unique compared to our previous reports of medicinal herbs that selected for deeper representation of species belonging to a shared genus [10]. Among taxa induced by all herbs, the* Klebsiella *spp. (3) and* Enterobacter *spp. (2) genomes encode broad sugar utilization pathways whereas other taxa are assacharolytic or encode very limited sugar utilization potential such as* Eubacterium eligens, Acidaminococcus intestini, Lutispora thermophila, Sutterella massiliensis* (discussed below).

Reports detailing antimicrobial properties of ginger and turmeric [4, 8, 15] suggest that the modulatory capacity of these herbs may involve active inhibition of sensitive taxa. For example, we noted 40 taxa that displayed reduced relative abundance in all herb-supplemented cultures (Table S2C). Consistent with the profiles depicted in Table S2A, the depth of species representation of impacted genera is limited with the exception of widespread suppression of 5* Alistipes *spp. and 3* Eubacterium *spp. including* E. contortum, E. dolichum, *and* E. hallii *that displayed low relative abundance in control cultures were further reduced in medicinal herb cultures. Based on relative abundance measurements alone, we cannot discriminate whether such taxa are subject to direct repression or decreased fitness due to shifts in community-metabolism of herb-substrates.

We further analyzed taxa displaying strong reductions in relative abundance (>1000-fold) relative to control cultures (Table S2D). Six taxa were strongly inhibited by all herbs including* Lachnotalea glycerini, Phocea massiliensis, Odoribacter splanchinicus, Escherichia coli, Shigella dysenteriae, *and* Romboutsia sedimentorum*. Turmeric strongly inhibited 10 species, including* Citrobacter *spp. (3),* Alistipes *(2), and* Eubacterium *spp. (2). Ginger strongly inhibited 13 species without any obvious phylogenetic pattern. Pipli resulted in the strong decrease in relative abundance of 3 species. Black pepper did not uniquely inhibit any species observed.

**Figure 2:**
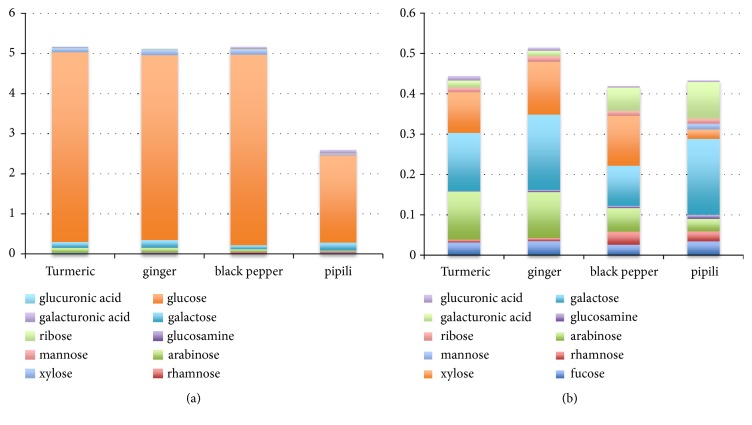
*Monosaccharide composition of medicinal herbs. *(a) Proportions of monosaccharides detected in medicinal herbs: glucuronic acid, galacturonic acid, xylose, mannose glucose, galactose, arabinose, ribose, glucosamine, fucose, and rhamnose. Fructose was not detected. (b) Data displayed without glucose.

*Sugar composition of medicinal herbs.* To enhance our ability to interpret the microbiota profiles induced by each herbal medicine, we used quantitative mass spectrometry (MS) to characterize the monosaccharide composition of each herb. For all herbs, glucose is the dominant monosaccharide and is present at an abundance ~10-times greater than all additional sugars combined (Figures 2(a) and 2(b)). The sugar content of pipli was about half that of other herbs tested, due to 50% reductions in glucose abundance comparatively.

*Sugar utilization capabilities of medicinal herb-supplemented communities.* Using a genomic approach, we performed metabolic reconstruction to predict sugar utilization phenotypes of 2,228 human gut genomes pertaining to the 11 herb monosaccharides detected (Figure S1). This pathway curation pertained to 195 of the 225 taxa observed in control and herb-supplemented communities (Table S3). We calculated a community phenotype index (CPI) for each herb-supplemented community to represent the overall capability of the community to utilize each sugar. The average CPI of communities were increased for several sugars, including glucose utilization in black pepper (p=0.002) and pipli (p=0.0001) cultures.

Turmeric supplemented cultures enriched for taxa capable of galactose utilization (p=0.03), whereas ginger (p<0.0001), black pepper (p=0.002), and pipli (p<0.0001) all enriched for taxa that can utilize mannose. Mannose was only detected in pipli. Black pepper (p=0.05) and pipli (p=0.04,) enriched for xylose utilizing taxa, whereas pipli (p=0.004) enriched for taxa capable of arabinose utilization. All herb-supplemented cultures and reduced taxa capable of utilizing ribose (p=0.01-<0.0001) and all except ginger reduced representation of glucosamine utilizing taxa (p=0.007-<0.0001). Caution should be applied in interpreting these enrichments since they did not correlate with sugar content distinctions measured for each herb.

**Figure 3:**
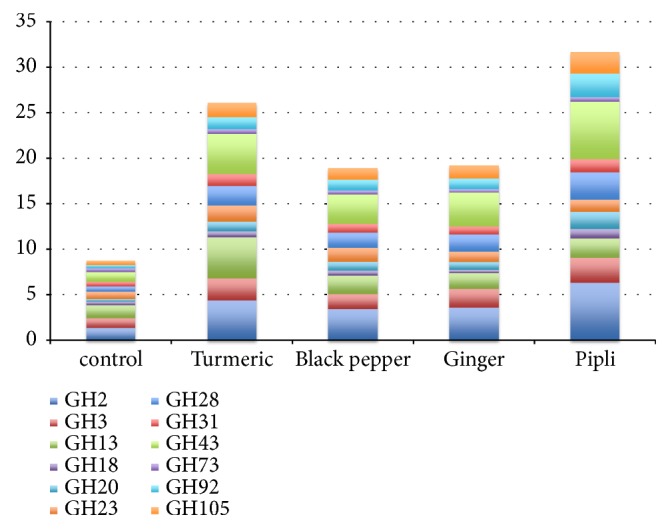
*Modulation of glycosyl hydrolase representation*. The relative abundance of taxa was multiplied by the number of genes in each GH family and summed for each herb.

*Glycosyl hydrolase representation is modulated by medicinal herbs.* We used the CAZy database to analyze glycosyl hydrolase (GH) gene families abundance in profiled taxa. Data was available for 63 taxa observed in cultures. The relative abundance of each taxon was multiplied by the number of encoded GH loci for each family. The relative abundance of GH family representation was substantially increased by all medicinal herbs compared to control cultures (Figure 3, Table S4). These alterations were particularly strong for turmeric (p=0.02, p<0.0001) and pipli (p=0.008, p=0.001), both impacting GH2 and GH43, whereas black pepper (p=0.0006) and ginger (p<0.0001) only influenced GH43 representation, whereas turmeric (p=0.004) strongly increased GH13. Pipli-supplemented cultures increased the representation of GH20 (p=0.006) and finally turmeric (p=0.007) and black pepper (p=0.02) increased the representation of GH28. Despite the unique taxonomic profiles of black pepper and ginger supplemented cultures, their impact on GH family representation was remarkably similar. These results suggest that the herbs tested impacted the growth of taxa encoding large repertoires of GH specificities and select for their proportions dependent on the specific glycans present in each herb.

*Amino acid metabolizing taxa are repressed by the medicinal herbs.* We compared the 55 most abundant taxa in control cultures (>0.01%). This portion of the community is expected to be enriched in taxa that generate energy by amino acid fermentation (Table S5). Several taxa displaying high relative abundance in control cultures were unaltered by herb-supplementation, suggesting that these taxa maintain their metabolic preferences in herb-supplemented cultures. With few exceptions, the remaining taxa displayed reduced abundance in herb-supplemented cultures. Among the few exceptions are taxa that encode a large repertoire of sugar utilization pathways such as* Enterococcus cloacae, Sutterella wadsworthensis, *and* B. vulgatus *that were increased by all herbs tested. The most profound change involved* Romboutsia sedimentorum *(10% in control cultures) which decreased to 0.3% in black pepper-supplemented cultures and was below detection in remaining herb-supplemented cultures. Similarly,* Oscillibacter valericigenes *(2.7% in control cultures) was decreased below detection in black pepper and pipli-supplemented cultures. Finally ginger-supplemented cultures reduced the relative abundance of* Bacteroides nordii *and* Dorea formicigenerans *(1.9% and 1.1% in control cultures, respectively) to below detection. Collectively, these results suggest that compared to control cultures, herb-supplemented cultures underwent a shift from amino acid fermentation to sugar fermentation that negatively impacted the relative fitness of amino acid fermenters.

*Turmeric-supplemented cultures are predicted to increase butyrate-producing taxa.* We used available reference bacterial genomes to reconstruct butyrate and propionate biosynthesis pathways encoded within medicinal herb-supplemented communities. Four alternative pathways for butyrate production were reconstructed that utilize the substrates acetyl-CoA, succinyl-CoA, L-glutamate, and L-lysine (Figure S1). For propionate synthesis, we analyzed 4 distinct biochemical pathway variants, namely, the propanediol, acrylate, succinate pathways, and the Wood-Werkman cycle, which ferments pyruvate to propionate using the modified succinate pathway and TCA cycle. These analyses resulted in the identification of a large number of taxa with SCFA biosynthetic potential, including 82 butyrate and 98 propionate producers (Table S6).

**Figure 4:**
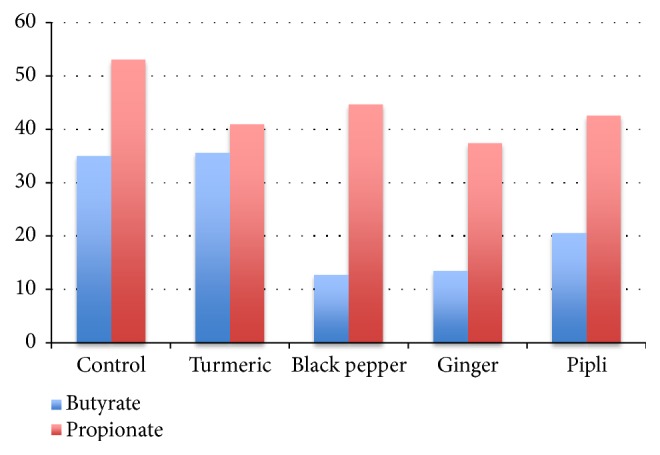
*Herb responsiveness of predicted butyrate and propionate producing taxa*. Presence (1) or absence (0) of butyrate biosynthetic pathways multiplied by relative abundance of taxa observed in each culture condition.

We analyzed the relative abundance of these taxa in response to herb-supplementation by calculating cumulative CPIs for butyrate and propionate in each culture condition as a prediction of relative butyrate and propionate production potential induced by each medicinal herb (Figure 4 and Table S6). Control cultures lacking carbohydrate produce butyrate* via *L-glutamate and L-lysine fermentation. By comparison to predictions of HMP 16S rDNA profiles of 400 fecal samples,] that estimate CPIs for butyrate average 25% (not shown), whereas control cultures are predicted to have average CPI=35%. Despite the elevated fraction of butyrate producers in control cultures, turmeric-supplemented cultures are predicted to have similar CPI values. The remaining medicinal herbs did not positively select for butyrate-producing taxa. Control cultures positively selected a number of abundant* Bacteroides *spp. that drive high CPI for propionate. All of the medicinal herbs tested are similarly predicted to select for a variety of propionate-producing taxa. By contrast, propionate-producing taxa in herb-supplemented cultures are not driven by* Bacteroides *spp. or any other dominant taxonomic group.

## 4. Discussion

Despite evidence that culinary herbs provide benefits to digestive health and immune homeostasis, investigations of these herbs are primarily focused on characterization of herb metabolites with drug-like effects. Studies recognizing the potential role of gut bacterial communities as potential mediators of host responses are currently lacking. To better understand the microbiological impact of medicinal herb substrates on complex fecal communities, we exploited* in vitro *anaerobic cultivation, which is free of complicating and poorly understood host-driven effects. The modulation of fecal communities by medicinal herbs is more complex than those selected by traditional prebiotic isolates that generally represent pure polysaccharide compounds (SNP, unpublished data). This observation is consistent with the greater diversity of substrates with prebiotic potential in herbs. Medicinal herbs also contain a large spectrum of phytonutrients, polyphenols, and diverse metabolites unique to each herb. Some herb metabolites are also expected to have inhibitory effects on sensitive taxa.

Analysis of culinary herb-supplemented communities indicated that turmeric, ginger, black pepper, and pipli all possess similar and substantial community modulatory potential (Figure 1(a)). Each herb selected for unique communities featuring both distinct expansions and contractions of several bacterial families (Figure 1(b)). We note that ginger strongly selected for members of* Coriobacteriaceae* that represent a diverse group of bacteria with isoflavone biotransformation [30], bile acid conversion [31], and generation of cholesterol-derived compounds [32]. With the exception of turmeric, all herbs resulted in blooms of beneficial* Bifidobacteriaceae*, whereas turmeric, black pepper, and, particularly, pipli induced an increase in relative abundance of* Bacteroidaceae *(Figure 1(c)). These taxonomic groups encode a high diversity and number of glycosyl hydrolase (GH) specificities required to catabolize diverse polysaccharide substrates. Turmeric-supplemented communities displayed an expansion of butyrate-producing* Lachnospiraceae*. This taxonomic group features a wide variety of sugar and amino acid fermenting taxa. These results highlight the medicinal herb-dependent restructuring of fecal communities and its relationship to potentially important functional shifts in community metabolism.

We noted that the modulatory signatures of culinary herbs differed from those documented for a group of 10 nervine medicinal herbs (manuscript in review). The nervine herbs selected for broader representation of species belonging to common genera. A potential interpretation of this difference is that culinary herbs contain less diversity in their glycan substrates compared to the nervine herbs. Among the 30 taxa displaying increased relative abundance in all herb-supplemented cultures is include enrichment of* Bacteroides *spp.,* Enterobacter *spp., and* Klebsiella *spp. (Table S2A). These results are consistent with our previous report of these taxa being increased in gut microbiota of human subjects following an 8-week turmeric intervention [8]. We noted that several taxa induced by all herbs in the current study encoded no or very limited sugar metabolism potential, whereas others are highly adapted to broad sugar utilization (Table S3). Amino acids are available in all culture media used and in control cultures represent the only substrate that can be used for energy production. The herb substrates responsible for the increased fitness of assacharolytic taxa remain unclear from our findings. All medicinal herb-supplemented cultures increased the relative abundance of a number of beneficial taxa including* Eubacterium rectale* [33, 34],* Gemmiger formicilis* [35], and* Bacteroides thetaiotaomicron* [36–38].

We noted a large number of taxa displaying reduced relative abundance in all or most of the medicinal herb-supplemented cultures (Table S2C). Various reports documenting antimicrobial activities associated with these herbs [3, 17, 39] suggest that some of the repressed taxa are susceptible to herb metabolites thus leading to growth inhibition. It is not possible to differentiate alternative explanations such as induced shifts in substrate metabolism. We speculate that both types of inhibition are operating in medicinal herb-supplemented communities. We analyzed taxa that were reduced by >1000-fold relative to control cultures (Table S2D). Six taxa were strongly inhibited by all herbal medicines tested, whereas others displayed reduced fitness in a subset of herbs or were uniquely impacted by a single herb. Taxa uniquely impacted by turmeric were the most prevalent. Interestingly, all culinary herbs analyzed resulted in reduced relative abundance of a number of pathogenic and opportunistic pathogens. It will be of interest to determine whether this effect is observed in human interventions. By contrast, all herbs positively selected for* Klebsiella pneumonia *and related species that may have pathogenic potential.

We measured the abundance of monosaccharides present in each medicinal herb with mass spectrometry (Figure 2). Glucose was the dominant sugar in all herbs. This composition bias was not the case for any of the nervine herbs we previously examined where glucose abundance was consistent with other detected monosaccharides (PLOS, in review). This result may explain the more restricted number of species belonging to common genera displaying increased fitness in culinary herb-supplemented cultures. Additional sugars were also detected in the spices including fucose, rhamnose, arabinose, glucosamine, galactose, xylose, mannose, ribose, galacturonic acid, and glucuronic acid. No fructose was detected in any spice, and mannose was only detected in pipli. We conclude that culinary herb glycans are enriched in glucose polymers that are complemented by additional sugars that individually are represented at abundances ranging from 7% to less than 0.1% with an average abundance of 0.8 - 1.6%.

We curated the sugar utilization pathways in a large fraction of genomes represented in the medicinal herb-supplemented fecal cultures (Table S3). We noted enrichment of several sugar metabolism pathways pertaining to sugars present in each herb; however, we were unable to find significant relationships between those sugar utilization pathways and the abundance of corresponding sugars. These particular findings suggest that sugar content alone is not a strong predictor of herb-dependent microbiota modulatory impact and that the specific sugar-linkages and branching characteristics may drive positive selection of microbial taxa.

To gain additional evidence that herb glycans uniquely drive aspects of the alterations in the gut microbial community structures that were observed, we analyzed the occurrence of GH families in available and pertinent reference genomes (Figure 3, Table S4). Our results indicate that each medicinal herb drives significant alterations in GH family representation and abundance compared to control cultures. The relative abundance of GH families is increased by all medicinal herbs compared to control cultures. Turmeric and pipli metabolism selected for the largest restructuring of GH families, particularly GH2 and GH43, whereas turmeric also strongly selected for GH13. These families encode GHs with broad substrate specificity and feature diverse glucose polymer degradation potential. Despite the unique taxonomic profiles of black pepper and ginger supplemented cultures, the impact on GH family representation is remarkably similar. This is consistent with the observed clustering of microbiota communities selected by these herbs (Figure 1). These results suggest that the medicinal herbs that were tested impacted the growth of microbial taxa encoding large repertoires of GH loci. The herb-dependent selection of specific GH functions suggests that herb glycans apply a selective pressure on fecal communities that also favor those taxa that can cross-feed on the liberated sugar moieties.

We noted that the most abundant taxa present in control cultures that lack carbohydrate energy sources but provided amino acids for energy production are nearly all reduced or remain unaltered in relative abundance in all herb-supplemented cultures (Table S5). Given that herb-supplemented media provide equivalent amino acid concentrations, the sweeping decreased fitness of taxa deriving energy from amino acid fermentation suggests that herb substrate utilization by fecal communities positively selects for carbohydrate utilizers at the expense of amino acid fermentation.

We curated butyrate and propionate biosynthetic potential of a large fraction of taxa present in culture (Table S6). All medicinal herbs modulated the relative abundance of many predicted butyrate- and propionate-producing taxa. With the exception of turmeric, which selected for increased butyrate production potential, the cumulative abundance of butyrate and propionate producers (community-wide) was not increased above that observed in control cultures (Figure 4). It should be noted that among the most abundant taxa in control cultures are 7 species predicted to produce butyrate* via *metabolism of L-glutamate and L-lysine, contributing to 19% of the total community, whereas herb-supplemented cultures select for butyrate producers that favor sugar fermentation. In this regard, the predicted butyrate biosynthetic potential of herb-supplemented cultures is compared to a standard that is 10% higher than the average predicted butyrate producers (25%) present in 400 human fecal microbiota (DAR, unpublished results).

Genome-based reconstruction of metabolic potential can be applied to a large number of pathways and functions encoded in gut microbiomes. Here we applied this approach to pathways and functions involved in degradation of glycan substrates, sugar utilization, and both butyrate and propionate biosynthetic potential to enhance the interpretation of microbial dynamics induced by culinary herbs. Our results suggest that complex, glucose-rich glycans present in herb-supplemented cultures provide a dominant selective pressure on gut communities* in vitro.* While the relative abundance of taxa predicted to produce butyrate and propionate was not further increased compared to control cultures, we observed a decrease in relative abundance in butyrate production* via *amino acid fermentation. These herbs negatively selected for amino acid fermenting taxa.

Compared to conventional single carbohydrate substrate prebiotics, medicinal herbs present a greater diversity of glycan substrates with commensurately larger effects on gut microbial community composition. In this regard, medicinal and culinary herbs represent an overlooked reservoir of dietary substances with potent prebiotic potential. Our data suggests that substrates present in culinary spices drive beneficial alterations in gut communities thereby altering their collective metabolism to contribute to the salubrious effects on digestive efficiency and health. Thus, further evaluation of these medicinal herbs on digestive health in human clinical trials is warranted and gut microbiota are a potentially important component and contributor to the health benefits of these herbs.

## Figures and Tables

**Table 1:** *Digestive herbal medicines examined in the current study*. Selected common names and family information are shown. *Curcuma longa* (common name: turmeric), *Zingiber officinale* (common name: ginger), *Piper longum* (common name: pipli or long pepper), and *Piper nigrum* (common name: black pepper).

Species	Common Name	Family
*Curcuma longa*	turmeric	Zingiberaceae
*Zingiber officinale*	ginger	Zingiberaceae
*Piper longum*	pipli or long pepper	Piperaceae
*Piper nigrum*	black pepper	Piperaceae

## Data Availability

The datasets generated for this study can be found in the NCBI GenBank database [BioProject ID PRJNA545727]. All datasets analyzed for this study are included in the manuscript and the supplementary materials.

## Supplementary Materials

Supplementary Materials Additional data referred to in the manuscript is included in the supplementary spreadsheets.* Supplemental Figure Legends. Figure S1. Reconstructed sugar transport and catabolic pathways in reference genomes.* Sugar utilization pathways for glucose, galactose, glucuronate and galacturonate, ribose, glucosamine, xylose, arabinose, rhamnose, fucose, and mannose. Four variants of butyrate biosynthesis using pyruvate, succinate, glutamate, or lysine. Four variants of propionate biosynthesis (P1-P4) using lactaldehyde/propanediol, lactate, or acetate.* Supplementary Table Legends. Table S1. 16S rRNA profiling data and statistical significance. A.* Relative abundance values of normalized 16S rRNA profiles.* B.* We applied Kruskal-Wallis test to provide evidence of a difference between the mean ranks of at least one pair of groups. Dunn's pairwise tests were carried out to examine our hypothesis and results were then adjusted using the Bonferroni correction.* Table S2. Medicinal herbs impact distinct microbes. A.* Taxa in each herb-selected community with a relative abundance >1% of the total.* B.* Taxa displaying increased average abundance >5-fold compared to control in all herb-supplemented cultures. Control values represent relative abundance and herb values are average fold-increase.* C.* Taxa displaying decreased average abundance >5-fold compared to controls in all herb-supplemented cultures.* D.* Taxa displaying >1000-fold decreased relative abundance in each herb-supplemented culture. Abundance values in bold represent statistically significant differences compared to control cultures.* Table S3. Sugar utilization pathways of bacterial taxa.* Taxa average fold-change (green>5-fold, yellow <5-fold, red> 5-fold, black=not detected). The presence or absence of sugar utilization pathways was scored at the species level as 1 or 0, respectively. When corresponding reference genomes were unavailable for specific taxa genus and family level assignments were predicted. In instances where multiple genomes were available and analyzed, fractional assignments were made depending on the conservation of sugar pathways. Taxa are sorted in descending order for sugar utilization pathways.* Table S4. Taxa encoding GH gene families.* Taxa observed in cultures, predicted to encode large number of GH, were used to determine the proportions of GH families present in each unique community. The number of genes encoding particular GH families was multiplied by the average relative abundance of each species representing an index of GH family representation for control and herb-supplemented communities.* Table S5. Putative amino acid fermenting taxa.* The most abundant taxa observed in control cultures lacking exogenous carbohydrate sources (green>5-fold, yellow <5-fold, red> 5-fold, black=not detected). Taxa producing butyrate from amino acid substrates. Abundance values in bold represent statistically significant differences compared to control cultures.* Table S6.* The presence or absence of butyrate and propionate pathways was scored at the species level as 1 or 0, respectively. When corresponding reference genomes were unavailable for specific taxa genus and family level assignments were predicted. In instances where multiple genomes were available and analyzed, fractional assignments were made depending on the conservation of SCFA pathways.
