# Supplementary figures and images for: Prebiotic Potential of Culinary Spices Used to Support Digestion and Bioabsorption
Source: Evid Based Complement Alternat Med. 2019 Jun 2;2019:8973704. doi: 10.1155/2019/8973704 (PMC6590564; doi:10.1155/2019/8973704)

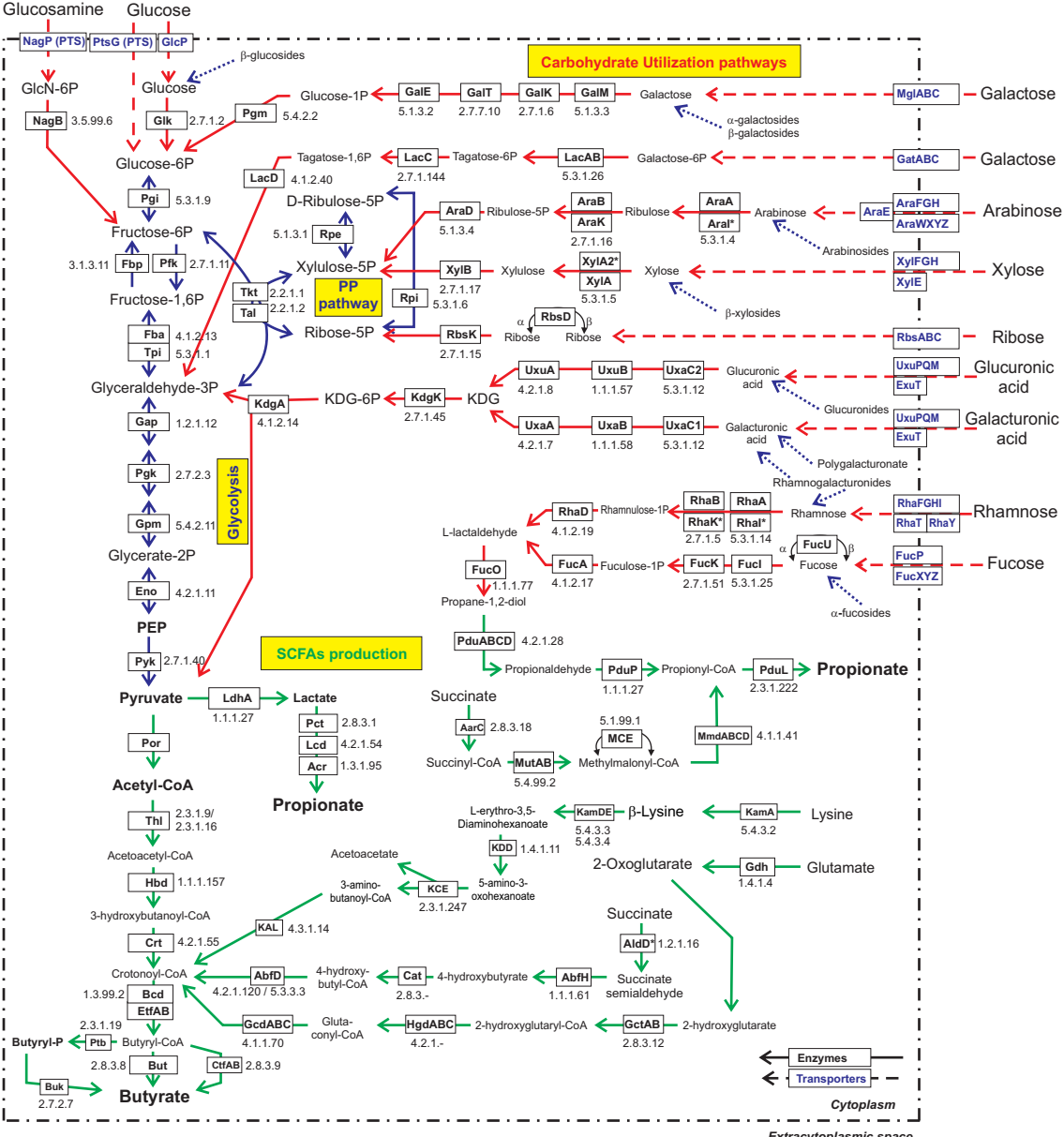

Supplement: Supplementary Materials — Additional data referred to in the manuscript is included in the supplementary spreadsheets. Supplemental Figure Legends. Figure S1. Reconstructed sugar transport and catabolic pathways in reference genomes. Sugar utilization pathways for glucose, galactose, glucuronate and galacturonate, ribose, glucosamine, xylose, arabinose, rhamnose, fucose, and mannose. Four variants of butyrate biosynthesis using pyruvate, succinate, glutamate, or lysine. Four variants of propionate biosynthesis (P1-P4) using lactaldehyde/propanediol, lactate, or acetate. Supplementary Table Legends. Table S1. 16S rRNA profiling data and statistical significance. A. Relative abundance values of normalized 16S rRNA profiles. B. We applied Kruskal-Wallis test to provide evidence of a difference between the mean ranks of at least one pair of groups. Dunn's pairwise tests were carried out to examine our hypothesis and results were then adjusted using the Bonferroni correction. Table S2. Medicinal herbs impact distinct microbes. A. Taxa in each herb-selected community with a relative abundance >1% of the total. B. Taxa displaying increased average abundance >5-fold compared to control in all herb-supplemented cultures. Control values represent relative abundance and herb values are average fold-increase. C. Taxa displaying decreased average abundance >5-fold compared to controls in all herb-supplemented cultures. D. Taxa displaying >1000-fold decreased relative abundance in each herb-supplemented culture. Abundance values in bold represent statistically significant differences compared to control cultures. Table S3. Sugar utilization pathways of bacterial taxa. Taxa average fold-change (green>5-fold, yellow <5-fold, red> 5-fold, black=not detected). The presence or absence of sugar utilization pathways was scored at the species level as 1 or 0, respectively. When corresponding reference genomes were unavailable for specific taxa genus and family level assignments were predicted. In instances where [file 8973704.f1.pdf]
